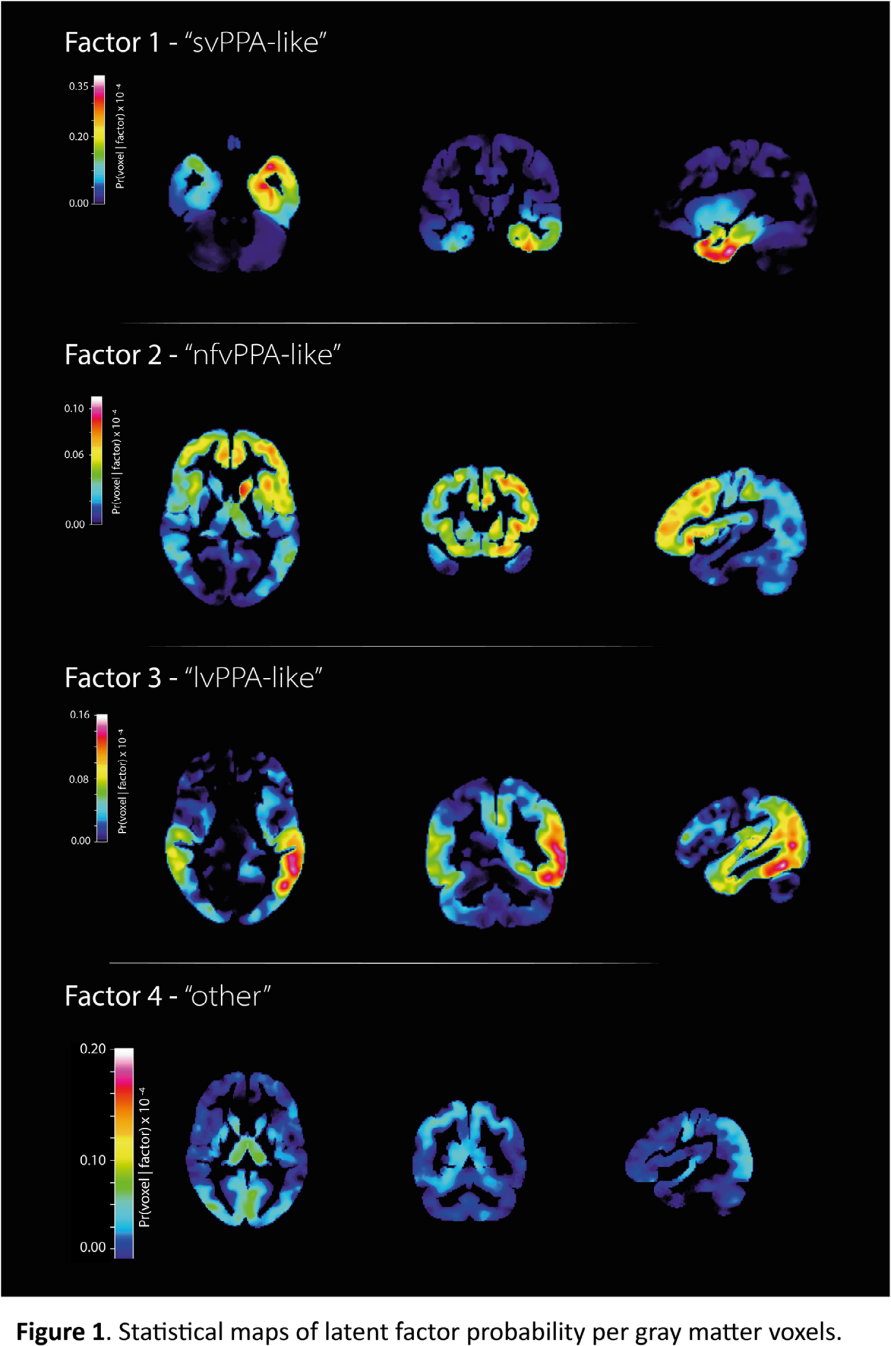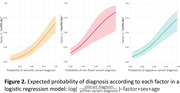# Latent atrophy factors in Primary Progressive Aphasia due to Alzheimer´s disease and linguistic features, across different cultural settings

**DOI:** 10.1002/alz.083973

**Published:** 2025-01-09

**Authors:** Ismael Luis Calandri, Colin Groot, Alex J. Wesseling, Florentina Morello Garcia, Ricardo Allegri, Frederik Barkhof, Laura E. Jonkman, Yolande A.L. Pijnenburg, Rik Ossenkoppele

**Affiliations:** ^1^ Alzheimer Center Amsterdam, Amsterdam UMC, Amsterdam Netherlands; ^2^ Fleni, Buenos Aires Argentina; ^3^ Alzheimer Center Amsterdam, Neurology, Vrije Universiteit Amsterdam, Amsterdam UMC location VUmc, Amsterdam Netherlands; ^4^ Alzheimer Center Amsterdam, Neurology, Amsterdam UMC, Amsterdam Netherlands; ^5^ Fleni, Buenos Aires, Buenos Aires Argentina; ^6^ Amsterdam Neuroscience, Brain Imaging, Amsterdam Netherlands; ^7^ Amsterdam UMC, location VUmc, Department of Anatomy and Neurosciences, Section Clinical Neuroanatomy and Biobanking, Amsterdam Netherlands; ^8^ Alzheimer Center Amsterdam, Neurology, Vrije Universiteit Amsterdam, Amsterdam UMC, Amsterdam Netherlands

## Abstract

**Background:**

Primary Progressive Aphasia (PPA) is a clinical entity that encompasses various neurodegenerative etiologies. Based on differential linguistic profiles, PPAhas been classified into three phenotypes (i.e., semantic [svPPA], non‐fluent [nfvPPA] and logopenic [lvPPA]), each characterized by unique atrophy patterns. Linguistic classification encounters a cultural barrier, as, e.g., certain features (like surface dyslexia) are difficult to objectify across languages, limiting the classification's generalizability. Our aim is to create an atrophy pattern detection model to predict PPA subtypes, independent of the cultural context that modifies linguistic features.

**Methods:**

We extract latent gray matter atrophy factors (LF) in a cohort of 134 Dutch‐speaking individuals with a clinical diagnosis of PPA. We use a data‐driven Bayesian modeling framework using latent Dirichlet allocation, and uncovered four LF’s from standardized gray matter density images (adjusted for age, sex, and MRI scanner). For each subject, the probability of belonging to each of the four LF’s was calculated. The probability assignment to each LF was compared against the linguistic phenotype assigned by a neurologist and AD biomarker status. The model was also applied to post‐mortem MRI data from 4 subjects to examine their neuropathological correlates.

**Results:**

the 4 LF’s are shown in Figure 1. LF1 (“svPPA‐like”) showed left‐predominant temporo‐polar atrophy; LF2 (“nfvPPA‐like”) left‐predominant fronto‐opercular and insular atrophy; LF3 (“lvPPA‐like") left‐predominant posterior‐parietal involvement; LF4 (“other”) parieto‐occipital. Higher LF1 probability was significantly associated with a clinical svPPA diagnosis (non‐standarized b=11, 95%CI[7.2‐15.0], p<0.001), higher LF2probability with a nfvPPA diagnosis (7.2[4.0‐11.0], p<0.001), and higher LF3 probability with lvPPA (9.0[5.9‐13], p<0.001). Higher LF3 probability was associated with a higher frequency of positive AD biomarkers (7[4.6‐11], p<0.001; Figure 2). Model generalizability will be tested by classifying 35 Spanish‐speaking subjects with PPA from Argentina based on their atrophy pattern (data presented at the conference).

**Conclusion:**

Our model demonstrates a strong ability to predict phenotypic classification and AD pathology through MRI. It can contribute to standardizing diagnostic performance across different cultural settings.